# Molecular characteristics of antibiotic-resistant *Escherichia coli* and *Klebsiella pneumoniae* strains isolated from hospitalized patients in Tehran, Iran

**DOI:** 10.1186/s12941-021-00437-8

**Published:** 2021-04-27

**Authors:** Javad Yasbolaghi Sharahi, Ali Hashemi, Abdollah Ardebili, Sara Davoudabadi

**Affiliations:** 1 Department of Microbiology, School of Medicine, Shahid Beheshti University of Medical Sciences, Tehran, Iran; 2 Infectious Disease Research Center, Golestan University of Medical Sciences, Gorgan, Iran; 3 Department of Microbiology, Faculty of Medicine, Golestan University of Medical Sciences, Gorgan, Iran

**Keywords:** *Klebsiella pneumoniae*, *Escherichia coli*, Antibiotic resistance genes, Carbapenem, Colistin

## Abstract

**Background:**

We evaluated the distribution of carbapenem and colistin resistance mechanisms of clinical *E. coli* and *K. pneumoniae* isolates from Iran.

**Methods:**

165 non-duplicate non-consecutive isolates of *K. pneumoniae* and *E. coli* were collected from hospitalized patients admitted to Iran's tertiary care hospitals from September 2016 to August 2018. The isolates were cultured from different clinical specimens, including wound, urine, blood, and tracheal aspirates. Antibiotic susceptibility testing was performed by disc diffusion and microdilution method according to the Clinical and Laboratory Standards Institute (CLSI) guideline. The presence of extended spectrum β-lactamases (ESBLs) genes, carbapenemase genes, as well as fosfomycin resistance genes, and colistin resistance genes was also examined by PCR-sequencing. The ability of biofilm formation was assessed with crystal violet staining method. The expression of colistin resistance genes were measured by quantitative reverse transcription-PCR (RT-qPCR) analysis to evaluate the association between gene upregulation and colistin resistance. Genotyping was performed using the multi-locus sequencing typing (MLST).

**Results:**

Colistin and tigecycline were the most effective antimicrobial agents with 90.3% and 82.4% susceptibility. Notably, 16 (9.7%) isolates showed resistance to colistin. Overall, 33 (20%), 31 (18.8%), and 95 (57.6%) isolates were categorized as strong, moderate, and weak biofilm-producer, respectively. Additionally, *bla*_TEM_, *bla*_SHV_, *bla*_CTX-M_, *bla*_NDM-1_, *bla*_OXA-48-like_ and *bla*_NDM-6_ resistance genes were detected in 98 (59.4%), 54 (32.7%), 77 (46.7%), 3 (1.8%), 17 (10.30%) and 3 (1.8%) isolates, respectively. Inactivation of *mgrB* gene due to nonsense mutations and insertion of IS elements was observed in 6 colistin resistant isolates. Colistin resistance was found to be linked to upregulation of *pmrA*-*C, pmrK, phoP*, and *phoQ* genes. Three of *bla*_NDM-1_ and 3 of *bla*_NDM-6_ variants were found to be carried by IncL/M and IncF plasmid, respectively. MLST revealed that *bla*_NDM_ positive isolates were clonally related and belonged to three distinct clonal complexes, including ST147, ST15 and ST3299.

**Conclusions:**

The large-scale surveillance and effective infection control measures are also urgently needed to prevent the outbreak of diverse carbapenem- and colistin-resistant isolates in the future.

## Background

*Enterobacteriaceae* are opportunistic pathogens that cause severe nosocomial infections, including urinary tract infections (UTIs), bloodstream infections, abdominal infections, and ventilator-associated pneumonia [1, 2]. *Escherichia coli* and *Klebsiella pneumoniae* are two important members of *Enterobacteriaceae* that have the ability to develop resistance to various classes of antibiotics. Nowadays, carbapenem antibiotics are recommended as the last-line therapy for MDR strains of *K. pneumoniae* and *E. coli* infections [1, 3]. However, increasing rate of resistance to carbapenems has complicated the treatment process and led to untreatable hospital infections [1, 4]. Resistance to carbapenems in *Enterobacteriaceae* is mainly mediated by the production of carbapenem-hydrolyzing enzymes (carbapenemases), among which *Klebsiella pneumoniae* carbapenemase (KPC), metallo-β-lactamases (VIM, IMP, NDM), and OXA-48 type of enzymes are the most common. Mobile genetic elements, including plasmids, transposons, and integrons are involved in the dissemination of related encoding genes [5–7].

New Delhi metallo-β-lactamase-1 (NDM-1) is one of the most important type of carbapenemases in carbapenem-resistant *Enterobacteriaceae* (CRE) [8, 9]. The *bla*_NDM_-positive strains are usually resistant to most antimicrobial agents in addition to β-lactams due to the co-existence of other resistance mechanisms [10]. Such resistant strains have known as the leading cause of infections associated with high mortality worldwide, representing a significant challenge for clinical management and public health [11]. Under these conditions, clinicians rely on a few alternative antibiotics e.g., colistin, fosfomycin, and tigecycline to treat infections caused by CRE [1, 12].

The old polymyxin antibiotic colistin (i.e., polymyxin E) is now recommended as the last choice for treatment of MDR Gram-negative bacteria, especially CRE infections [13]. The recent increase in the use of colistin in clinical practice, accompanied by its unbridled use in agriculture, have contributed to the rapid dissemination of resistance [14]. Colistin resistance is caused by decreases in the net negative charge of the outer membrane, loss of lipid A, or efflux pumps and plasmid-encoded *mcr* genes [15]. The *mcr-1* gene uses a target site modification mechanism to protect bacteria from the action of colistin. The *mcr* gene is observed on transferable plasmid and encodes an enzyme called phosphatidylethanolamine transferase which transfers the phosphatidylethanolamine residue to lipid A [16].

The main purpose of this study was to evaluate the antimicrobial resistance patterns and molecular mechanisms of carbapenem and colistin resistance among the clinical isolates of *E. coli and K. pneumoniae* from hospitalized patients admitted to tertiary care hospitals in Tehran, Ahwaz, Kashan, Tabriz, Sari, Gorgan, Birjand and Babol. In addition, the ability of biofilm production as well as clonal and genetic diversity of isolates were examined.

## Methods

### Ethical statement

This study was approved by the Ethics Committee of Shahid Beheshti University of Medical Sciences “IR.SBMU.MSP.REC.1397. 629”. In order to maintain patients confidentiality participants were anonymous and no personal information was collected or included in the study.

### Bacterial isolates

*K. pneumoniae* and *E. coli* isolates were collected from hospitalized patients infected in Iran hospitals from September 2016 to August 2018. The isolates were cultured from different clinical specimens, including wound, urine, blood, and tracheal aspirates. Each isolate was identified at species level based on the biochemical reactions, including reaction on SH2/indole/motility (SIM) medium, triple sugar iron (TSI) agar, urease production on urea agar, growth on Simmons'citrate agar medium, methyl red/Vogues-Proskauer (MR/VP), and ornithine decarboxylase (OD) test [17]. All isolates were stored in tryptic soy broth (TSB) tube with 20% glycerol at − 70 °C.

### Antimicrobial susceptibility testing

Antimicrobial susceptibility of all *E. coli* and *K. pneumoniae* isolates was determined by the Kirby-Bauer disk diffusion method on Cation-Adjusted Mueller Hinton agar (Merck, Germany) and interpreted as recommended by the Clinical and Laboratory Standards Institute (2018 CLSI breakpoints) or Food and Drug Administration (FDA) breakpoints guidelines (for tigecycline) [18, 19]. Antibiotic discs used were as follow: penicillins [piperacillin (PIP, 100 μg)], β-lactam/β-lactamase inhibitor combinations [piperacillin/tazobactam (PTZ, 100/10 μg)], cephems [ceftazidime (CAZ, 30 μg), cefotaxime (CTX, 30 μg), cefepime (FEP, 30 μg), cefpodoxime (CPD, 30 μg)], monobactams [aztreonam (ATM, 30 μg)], carbapenems [imipenem (IPM, 10 μg), meropenem (MEM, 10 μg), ertapenem (ETP, 10 μg), doripenem (DOR, 10 μg)], aminoglicosides [gentamicin(GEN,10 μg)], Amikacin (AK, 30 μg)], Fluoroquinolones [ciprofloxacin (CIP, 5 μg)], inhibitors [trimethoprim-sulfamethoxazole (TS, 2.5 μg)], fosfomycins [fosfomycin/trometamol (FOT, 200 μg)], tigecycline (TGC, 15 μg), and nalidixic acid (NA, 30 μg), (Mast Group, Merseyside, UK). The minimum inhibitory concentrations (MICs) of seven antibiotics, including imipenem, meropenem, ceftazidime, cefotaxime, cefepime, ciprofloxacin, and colistin were determined by broth microdilution method on Cation-Adjusted Mueller Hinton broth (Merck, Germany), and the results were analyzed according to the CLSI guidelines [18]. The 2016 EUCAST breakpoints were used (available at http://www.eucast.org/clinical_breakpoints/) for colistin. The antibiotic powders were purchased from Sigma-Aldrich (St. Louis, MO, USA)*. E. coli* ATCC 25922 was used as a quality control strain for disk diffusion and MIC results.

The CDC and the European Centre for Disease Prevention and Control (ECDC) have jointly developed definitions for multidrug-resistant (MDR), extensively drug-resistant (XDR) and pandrug-resistant (PDR) bacteria. MDR was defined as acquired non-susceptibility to at least one agent in three or more antimicrobial categories, XDR was defined as non-susceptibility to at least one agent in all but two or fewer antimicrobial categories and PDR was defined as non-susceptibility to all agents in all antimicrobial categories.

### Phenotypic detection of β-lactamases

Detection of ESBLs was tested for all the isolates by combination disc diffusion test (CDDT) containing ceftazidime (CAZ) and cefotaxime (CTX) with CAZ 30 μg + clavulanic acid 10 μg and CTX 30 μg + clavulanic acid 10 μg per disc (Mast Group, Merseyside, UK). *K. pneumoniae* ATCC 700,603 and *E. coli* ATCC 25,922 were used as positive and negative controls for ESBL production, respectively [22].

### Phenotypic detection of metallo β-lactamases

Combined disk diffusion test (CDDT) was performed for identification of MBLs by imipenem and meropenem (Mast Group, Merseyside, UK) alone and in combination with EDTA [20]. *Pseudomonas aeruginosa* ATCC 27853 and *P. aeruginosa* PA40 (Accession number: KM359725) were used as negative and positive controls for MBL production, respectively.

### Screening for carbapenemase production

The Carba NP test was performed for the detection of carbapenemase activity in isolates as described previously [21, 22].

### Biofilm formation assay

Assessment of biofilm formation was performed by the colorimetric microtiter plate assay in triplicates [20, 21]. Overnight cultures of bacterial isolates were suspended in tryptic soy broth (TSB) (Merck-Germany) at 37 °C. Then, 200 μL bacterial suspension with turbidity of 0.5 McFarland standard were transferred into the sterile 96-well polystyrene microplates (JET Biofil, Guangzhou, China). TSB without bacteria was used as negative control. After 24 h of incubation at 37 °C, each well was rinsed three times with phosphate buffered saline (PBS, pH 7.3) to remove any non-adherent cells. Fixation and staining the adherent cells were performed by methanol and 1% crystal violet (Merck, Germany). Then, plates were gently rinsed off with PBS and destained by 33% glacial acetic acid and finally OD of each well were measured at 492 nm. The criteria for categorization of isolates were including: strong biofilm producer (4 × ODc < OD), moderate biofilm producer (2 × ODc < OD < 4 × ODc), weak biofilm producer (ODc < OD < 2 × ODc) and no biofilm producer (OD < ODc) [23, 24].

### Detection of resistance genes

DNA was extracted using the DNA extraction kit (High Pure PCR Template Preparation Kit-Roche, Germany, Lot. No. 10362400) according to the manufacturer's instruction. Detection of resistance genes among all isolates, including ESBL-encoding genes (*bla*_TEM_, *bla*_SHV_, *bla*_CTX-M_, *bla*_GES_, *bla*_PER_, and *bla*_VEB_), carbapenemases genes (*bla*_OXA-48_, *bla*_NDM_, *bla*_KPC_, *bla*_VIM_, and *bla*_IMP_), and two fosfomycin resistance genes (*fosA* and *fosC2*), was performed by polymerase chain reaction (PCR) amplification using the specific primers [25–29] and confirmed by sequencing. *P. aeruginosa* containing *bla*_*GES*_*, bla*_*PER*_*, bla*_*VEB*_, *bla*_VIM_, *bla*_IMP_ genes and *K. pneumoniae* containing other genes received from Shahid Beheshti University of Medical Sciences, Tehran, Iran, were used as positive controls. PCR products were purified using a PCR purification Kit (Bioneer Co., Korea) and then, nucleotide sequencing of amplicons was performed by an ABI PRISM 3700 sequencer (Macrogen Co., Korea). Nucleotide sequences were analyzed using Chromas software version 1.45 (http://www.technelysium.com.au) and NCBI BLAST program (https://blast.ncbi.nlm.nih.gov/Blast.cgi).

### Molecular analysis of colistin resistance

Analysis of plasmid-mediated colistin resistance was performed by PCR amplification of *mcr-1*, *mcr-*2, *mcr-3*, and *mcr-4* among all colistin-resistant *K. pneumoniae* isolates. All colistin-resistant *K. pneumoniae* isolates were also examined for the presence of mutations in the chromosomally-encoded modifications of the LPS, including *mgrB*, *pmrA*, *pmrB*, *phoP*, and *phoQ* genes [30, 31]. Insertion sequences (ISs) were identified using the IS finder tool (https://www-is.biotoul.fr/index.php). Genomic DNA from two colistin-sensitive *K. pneumoniae* clinical isolates and *K. pneumoniae* ATCC 700603 were used as control.

### Real-time quantitative reverse transcription PCR

Colistin-resistant isolates were assessed for expression of *pmrC*, *pmrA*, *pmrB*, *pmrD*, *pmrE*, and *pmrK* genes using specific primers [29, 31, 32]. *rpsL* gene encoding a ribosomal protein was used as housekeeping gene to normalize the levels of transcripts tested. Total RNA was extracted from the cultures grown in the mid-log phase of growth in Luria–Bertani broth (Merck, Darmstadt, Germany) by the RNX-Plus Kit (Cat. No., RN7713C, Sinaclon, Iran) according to the manufacturer’s instruction. The contaminating DNA was removed by RNase-free DNase I (Fermentas, Thermo Fisher Scientific Inc., USA). The total RNA concentration was determined by Nanodrop (WPA Biowave II Nanospectrophotometer, USA). DNase-treated RNA was reverse-transcribed into cDNA using the Takara Kit (Japan). RNA samples were checked for contaminating DNA by PCR. Real-time PCR assay was performed on synthesized cDNA using the Power SYBR Green PCR Master Mix (Bioneer, Korea) on a Corbett Rotor-Gene 6000 real-time rotary analyzer (Corbett Life Science, Australia). Each amplification protocol included a first denaturation step of 10 min at 94 °C, followed by 40 cycles of 20 s at 94 °C and 45 s at 59 °C. All samples were run in triplicate. Data were compared to those obtained with the *rpsL* gene. The expression level of transcripts was calculated based on 2^−ΔΔCT^ method (relative) against that for the susceptible isolate, *K. pneumoniae* ATCC 700603. Experiments were repeated three times. The parameter Ct was defined as the threshold cycle number at which the first detectable fluorescence generated by the binding of SYBR Green I dye to the minor groove of double-stranded DNA began to increase exponentially.

### Plasmid manipulation and analysis

NDM positive strains were selected for plasmid analysis. Plasmid DNA of isolates, transconjugants, and transformants was extracted by using the Roche kit (Cat. No. 11 754 777 001) according to the manufacturer’s instructions. Electroporation was used to transform plasmids encoding *bla*_NDM_ into *E. coli* TOP10. The *bla*_NDM_ transformants were selected on MH agar (Merck-Germany) supplemented with meropenem (0.5 mg/L) (Sigma–Aldrich). Conjugation experiments were carried out in LB broth with sodium-azide-resistant *E. coli* J53AzR as the recipient. Cultures of donor and recipient cells in logarithmic phase were added to 4 mL of fresh LB broth and were then incubated at 37 °C overnight without shaking. The transconjugants were selected on MH agar (Merck-Germany) supplemented with meropenem (0.5 mg/L) or ceftazidime (1, 2 and 4 mg/L) with sodium azide (100 mg/L) (Sigma–Aldrich).

### PCR-based replicon typing

All transconjugants and transformants were typed by a PCR method based on replicons of the major plasmid incompatibility groups among *Enterobacteriaceae* [33].

### Multi-locus sequence type (MLST) analysis

Genotyping by MLST analysis was conducted to characterize diversity and epidemiology of *bla*_NDM_- carrying *K. pneumoniae* isolates [34]. Briefly, PCR for seven housekeeping genes, including *rpoB*, *gapA*, *mdh*, *phoE*, *pgi*, *infB*, and *tonB* was carried out. Results were analyzed according to the Institute Pasteur *Klebsiella* MLST database (https://bigsdb.pasteur.fr/klebsiella/klebsiella.html). Unique sequence (allele) number for each gene was assigned on the basis of the information in the *K. pneumoniae* MLST database to determine specific sequence types (ST). A combination of the allelic sequences of the seven genes yielded the allelic profile for each isolate.

### Repetitive extragenic palindromic (rep)-PCR typing

Rep-PCR analyses were conducted with the single primer BoxA1R (5′-CTA CGG CAA GGC GAC GCT GAC G-3′) [35]. To determine phylogenetic relationships, rep-PCR profiles were analyzed by GelCompar II software (Applied Maths, Belgium) using the Pearson’s correlation coefficient with unweighted paired group method using arithmetic averages (UPGMA) as well as at the 80% similarity level [35].

### Statistical analysis

Chi-squared test was performed using SPSS software, 21.0 (SPSS Inc., Chicago, IL, USA) to check for any significant differences between datasets. A significant level of *P* ≤ *0.05* was considered statistically significant.

## Results

### Bacterial isolates

165 non-duplicate non-consecutive isolates of *E. coli* and *K. pneumoniae* were collected from 73 (45.5%) females and 92 (54.5%) males admitted at five Iranian hospitals during the September 2016 to August 2018. The age range of patients was between 1 and 87 years. The origins of isolates were 114 in urine, 39 in tracheal aspirates, 4 in wounds, and 8 in blood.

### Antimicrobial susceptibility

Antibiotic resistance patterns of 165 isolates of *K. pneumoniae* and *E. coli* are shown in Table 1. The lowest rate of resistance was observed against tigecycline (n = 9, 5.5%), and fosfomycin (n = 26, 15.8%). The number of isolates with multidrug-resistant (MDR), extensively drug-resistant (XDR), and pandrug-resistant (PDR) phenotype was 32 (*E. coli*: 27, *K. pneumoniae*: 5), 120 (*E. coli*: 77, *K. pneumoniae*: 43), 1 (*K. pneumoniae*: 1), respectively. The MIC ranges, MIC_50_, MIC_90_, and the percentages of isolates resistant, intermediate, or susceptible isolates to the seven antimicrobial agents are shown in Table 2.

**Table 1:** Antibiotic resistance patterns of 165 isolates of *K. pneumoniae* and *E.* coli

Species (no (%) of isolates)	Antibiotic resistance patterns	ATM	GM	CIP	TS	AK	CTX	CAZ	FEP	NA	PIP	TGC	DOR	ETP	IMI	MEM	PTZ	FOT
*E. coli* (113)	Susceptible	18 (15.9%)	65 (57.5%)	5 (4.4%)	16 (14.2%)	54 (47.8%)	0	7 (6.2%)	6 (5.3%)	2 (1.8%)	1 (0.9%)	104 (92%)	56 (49.6%)	49 (43.4%)	83 (73.5%)	70 (61.9%)	59 (52.2%)	94 (83.2%)
	Intermediate	17 (15%)	12 (10.6%)	13 (11.5%)	1 (0.9%)	37 (32.7%)	9 (8%)	7 (6.2%)	11 (9.7%)	6 (5.3%)	2 (1.8%)	6 (5.3%)	35 (31%)	32 (28.3%)	18 (15.9%)	21 (18.6%)	28 (24.8%)	7 (6.2%)
	Resistant	78 (69%)	36 (31.9%)	95 (84.1%)	96 (85%)	22 (19.5%)	104 (92%)	99 (87.6%)	96 (85%)	105 (92.9%)	110 (97.3%)	3 (2.7%)	22 (19.5%)	32 (28.3%)	12 (10.6%)	22 (19.5%)	26 (23%)	12 (10.6%)
*K. pneumoniae* (52)	Susceptible	6 (11.5%)	10 (19.2%)	3 (5.8%)	4 (7.7%)	13 (25%)	0	1 (1.9%)	1 (1.9%)	0	0	32 (61.5%)	7 (13.5%)	12 (23.1%)	13 (25%)	20 (38.5%)	9 (17.3%)	36 (69.2%)
	Intermediate	12 (23.1%)	8 (15.4%)	4 (7.7%)	0	4 (7.7%)	3 (5.8%)	2 (3.8%)	15 (28.8%)	3 (5.8%)	1 (1.9%)	14 (26.9%)	7 (13.5%)	3 (5.8%)	3 (5.8%)	0	7 (13.5%)	2 (3.8%)
	Resistant	34 (65.4%)	34 (65.4%)	45 (86.5%)	48 (92.3%)	35 (67.3%)	49 (94.2%)	49 (94.2%)	36 (69.2%)	49 (94.2%)	51 (98.1%)	6 (11.5%)	38 (73.1%)	37 (71.2%)	36 (69.2%)	32 (61.5%)	36 (69.2%)	14 (26.9%)

*ATM* aztreonam, *GM* gentamicin, *CIP* ciprofloxacin, *TS* trimethoprim-sulfamethoxazole, *AK* Amikacin, *CTX* cefotaxime, *CAZ* ceftazidime, *FEP* cefepime, *NA* nalidixic acid, *PIP* piperacillin, *TGC* tigecycline, *DOR* doripenem, *ETP* ertapenem, *IMI* imipenem, *MEM* meropenem, *PTZ* piperacillin/tazobactam, *FOT* fosfomycins

**Table 2:** MIC of the *K. pneumoniae and* E. *coli* clinical isolates (n = 165)

Antibiotic	MIC (µg/mL)			No (%)		
	Range	50%	90%	Resistant	intermediate	susceptible
Ceftazidime	2– ≥ 512	64	512	153 (92.7%)	4 (2.42%)	8 (4.84%)
Cefotaxime	2– ≥ 512	64	512	153 (92.7%)	4 (2.42%)	8 (4.84%)
Cefepime	2–512	32	256	150 (90.9%)	0	15 (9.1%)
Ciprofloxacin	2–512	32	128	150 (90.9%)	0	15 (9.1%)
Imipenem	≤ 2–128	2	32	55 (33.3%)	10 (6%)	95 (57.6%)
Meropenem	≤ 2–256	2	32	55 (33.3%)	10 (6%)	95 (57.6%)
Colistin	0.25–128	0.5	4	16 (9.7%)	0	149 (90.3%)

K54 was found to be non-susceptible to all antibiotics tested, which includes cephalosporins, penicillins, carbapenems, aztreonam, aminoglycosides, ciprofloxacin, colistin, tetracyclines, tigecycline, trimethoprim-sulfamethoxazole and fosfomycin (Table 3). Thus, the isolate can truly be described as pandrug-resistant.

**Table 3:** MIC and molecular features related to NDM-producing and colistin-resistant *K. pneumoniae* isolates

Isolates	MIC (µg/mL)							ESBL genes	MBL genes	Sensitivity to antibiotic
	CIP	CTX	CEP	CAZ	IMI	MER	CO			
K37	128	64	64	64	128	128	128	CTX-M, TEM, SHV	–	FOS
K38	128	64	64	64	128	128	64	CTX-M, TEM	–	TGC, FOS
K50	256	512	512	512	8	8	64	CTX-M, TEM, SHV	–	TGC, FOS
K52	256	512	512	512	8	16	4	CTX-M	–	TGC
K53	128	512	512	512	8	8	4	CTX-M, SHV	–	TGC
K54^a^	256	128	128	128	32	16	16	CTX-M, TEM	–	–
K57	256	16	16	16	16	16	4	CTX-M, SHV	–	TGC
K83	128	32	32	32	32	8	128	CTX-M, TEM, SHV	–	TGC, FOS
K101	128	512	512	512	64	64	128	CTX-M,TEM, SHV	–	TGC, FOS
K111	16	128	128	128	8	16	4	CTX-M, TEM	–	TGE, FOS
K130	128	512	512	512	8	32	8	CTX-M. TEM, SHV	–	TGC
K134	16	64	64	64	8	8	4	CTX-M, TEM	–	FOS, TGE
K136	128	512	64	512	16	16	8	TEM, SHV	–	MER
K148	16	8	16	16	16	32	4	TEM	–	TGE
K151	16	16	16	16	4	4	4	TEM	–	DOR,TGE
K158	8	16	16	16	4	4	4	TEM	–	DOR, TGE, FOS
K36	128	32	32	32	128	128	1	CTX-M, TEM, SHV	NDM-1	CO
K72	128	32	32	32	32	64	1	CTX-M, TEM, SHV	NDM-1	CO, TGC, FOS
K120	128	512	512	512	16	16	1	CTX-M, TEM, SHV	NDM-1	CO
K161	128	32	32	32	128	32	1	CTX-M, TEM, SHV	NDM-6	CO
K162	64	64	64	64	64	64	1	CTX-M, TEM, SHV	NDM-6	CO
K165	128	64	64	64	64	64	1	CTX-M, TEM, SHV	NDM-6	CO

*CIP* ciprofloxacin, *CTX* cefotaxime, *CAZ* ceftazidime, *FEP* cefepime, *TGC* tigecycline, *DOR* doripenem, *ETP* ertapenem, *IMI* imipenem, *MEM* meropenem, *FOT* fosfomycin/trometamol, *CO* colistin

^a^Pandrug-resistant

### β-lactamase phenotype

The prevalence of ESBL-producing *E. coli* and *K. pneumoniae* was 49.6% (n = 82) and 26.6% (n = 44), respectively. The proportion of ESBL-producing *E.coli* and *K. pneumoniae* showing resistance to cephalosporin were significantly higher than non-ESBL-producing strains (*p* < 0.05).

### Metallo β-lactamase phenotype

The prevalence of MBL-producing *E. coli* and *K. pneumoniae* were 1.8% (n = 2) and 38.5% (n = 20), respectively. All MBL-producing isolates were resistant to carbapenems and cephalosporins (*P* ≤ 0.05).

### Carbapenemase phenotype

According to the results of the Carba NP test, only 22 K*. pneumoniae* isolates produced carbapenemase enzymes. As with the MBL phenotypes, all carbapenemase-producing isolates were resistant to carbapenem and cephalosporin antibiotics (*p* ≤ 0.05).

### Biofilm phenotype

Biofilm phenotype accounted for 159 out of 165 isolates (96.36%): 33 isolates (20%) produced strong biofilm, 31 isolates (18.8%) produced moderate biofilm, and 95 isolates (57.6%) produced weak biofilm; whereas 6 isolates (3.6%) did not form biofilm. Among 82 ESBL-producing *E. coli*, 12 (14.63%) isolates were strong biofilm-producers, 11(13.41%) were moderate biofilm-producers, 55 (67%) were weak biofilm-producers, and 4 (4.88%) isolates produced no biofilm. Moreover, among the 44 ESBL-producing *K. pneumoniae*, 16 (36.36%) isolates were strong biofilm-producers, 12 (27.27%) were moderate biofilm-producers, and 16 (36.36%) isolates were identified as weak biofilm-producers.

### Antimicrobial resistance genes

The prevalence of isolates carrying ESBL-encoding determinants was 78.2% (n = 129). The *bla*_TEM_, *bla*_SHV_, and *bla*_CTX-M_ genes were detected in 98 (59.4%), 54 (32.7%), and 77 (46.7%) isolates, respectively; while no isolates were positive for the *bla*_GES_, *bla*_PER_, and *bla*_VEB_ genes (Table 4). In addition, the prevalence of MBL-producing *E. coli* and *K. pneumoniae* were 1.8% (n = 2) and 38.5% (n = 20), respectively, of which 6 (6.5%) *K. pneumoniae* isolates were positive for for *bla*_NDM_ gene (*bla*_NDM-6_: 3, *bla*_NDM-1_: 3) (Table 4). No *bla*_IMP_, *bla*_VIM_, *bla*_SIM_, *bla*_GIM_, *bla*_SPM_, and *bla*_KPC_ genes were detected. The *bla*_OXA-48-like_ gene was identified among 17 (10.30%) of isolates. While no plasmid-mediated colistin resistance genes of *mcr-1, mcr-2, mcr-3, mcr-4*, and *mcr-4* were detected in isolates, 16 (9.7%) *K. pneumoniae* were identified as colistin-resistant*.* Moreover, the primers targeting *fosA* and *fosC2* genes did not provide any amplicon in fosfomycin-resistant isolates. The results from real-time PCR analysis were consistent with PCR and sequencing.

**Table 4:** Prevalence of beta-lactamase genes among isolates

No (%) of isolates	*bla*_TEM_	*bla*_SHV_	*bla*_CTXM_	*bla*_TEM_, *bla*_SHV_	*bla*_TEM,_ *bla*_CTXM_	*bla*_SHV_, *bla*_CTXM_	*bla*_TEM_, *bla*_SHV_, *bla*_CTXM_	*bla*_TEM_, *bla*_SHV_, *bla*_CTXM_, *bla*_NDM_
*E. coli* (n: 113)	19 (16.8%)	3 (2.6%)	16 (14.1%)	8 (7.1%)	19 (16.8%)	3 (2.6%)	12 (10.6%)	0
*K. pneumoniae* (n: 52)	13 (25%)	5 (9.6%)	1 (1.9%)	4 (7.7%)	7 (13.5)	3 (5.8%)	10 (19.2%)	6 (11.5%)

### Molecular analysis of colistin resistance

**Fig. 1:**
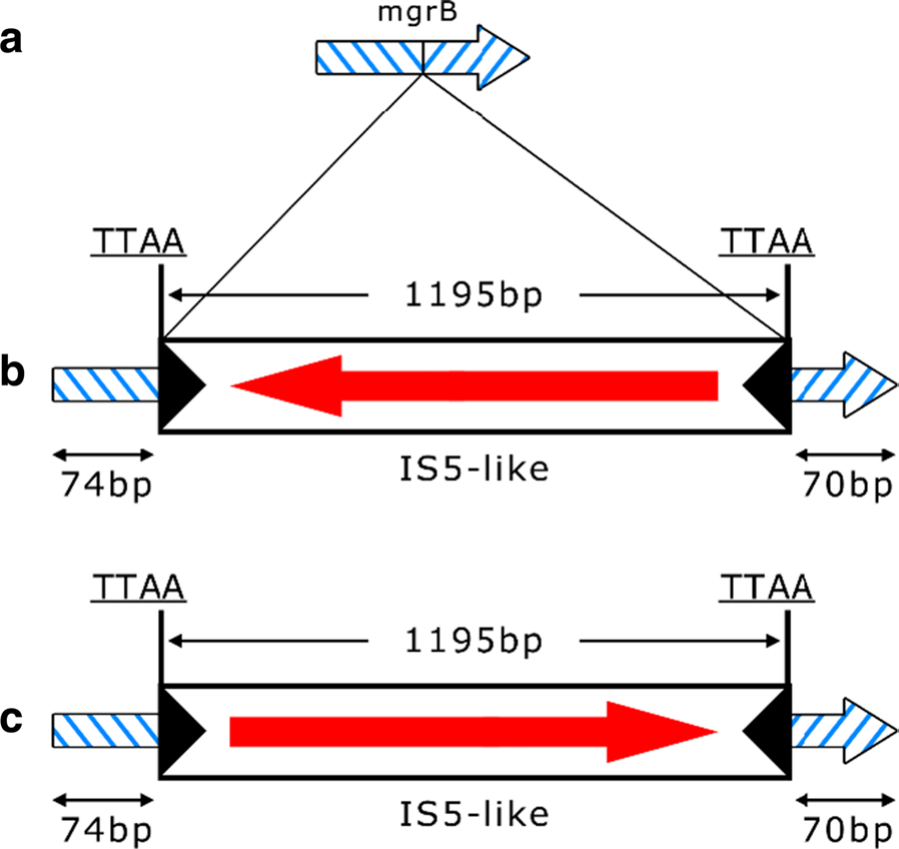
Schematic representation of the different insertion events identified in the *mgrB* gene. **a** The intact *mgrB* gene as found in wild type isolates and isolate (**b**) *mgrB* truncated by IS5-like in k37 isolate. **c** *mgrB* truncated by IS5-like as identified by Laurent Poirel et al.[65]

The *mcr-1*, *mcr-2*, *mcr-3*, and *mcr-4* genes were not found in any of the colistin-resistant isolates, we focused on other mechanisms of resistance, specifically *mgrB* gene inactivation and the presence of the mutations in the *pmrA*, *pmrB*, *phoP*, and *phoQ* genes. Sequence analysis of the *mgrB* gene showed that one isolate (K37) generated amplicon that was larger than those produced by *K. pneumoniae* K85 control isolate and colistin-susceptible *K. pneumoniae* ATCC 700603 strain. Amplicon sequencing revealed that insertional inactivation had occurred in the coding region of the *K. pneumoniae* K37 *mgrB* gene. Also, occurred at nucleotide 75 and was raised by insertional sequence that shared 99% identity at the nucleotide level with IS5 family of insertion sequences (Fig. 1). Insertional inactivation was not detected in other isolates tested. However, K83, K101, K50, and K130 isolates had premature amber stop codon (TAG) due to a C-to-T change at position 88 and K136 had premature opal stop codon (TGA) due to a C-to-A change at position 117, resulting in a truncated MgrB protein containing 29 and 39 amino acids, respectively. Amino acid substitutions were detected in PmrB, PhoP and PhoQ proteins. Nucleotide A at the position 469 of the *pmrB* gene was converted to C in K101 isolate, leading to Thr157Pro substitution**.** At nucleotide position of 171, the *phoP* gene underwent A to C conversion, resulting in single substitution Glu57Asp in the isolate K37. The isolate K83 showed nucleotide conversion A to G at the position 449 of *phoQ* gene, leading to substitution Asp150Gly. No amino acid substitutions were detected in PmrA protein.

### Overexpression of pmrCAB, pmrHFIJKLM, and phoPQ operons

**Fig. 2 a:**
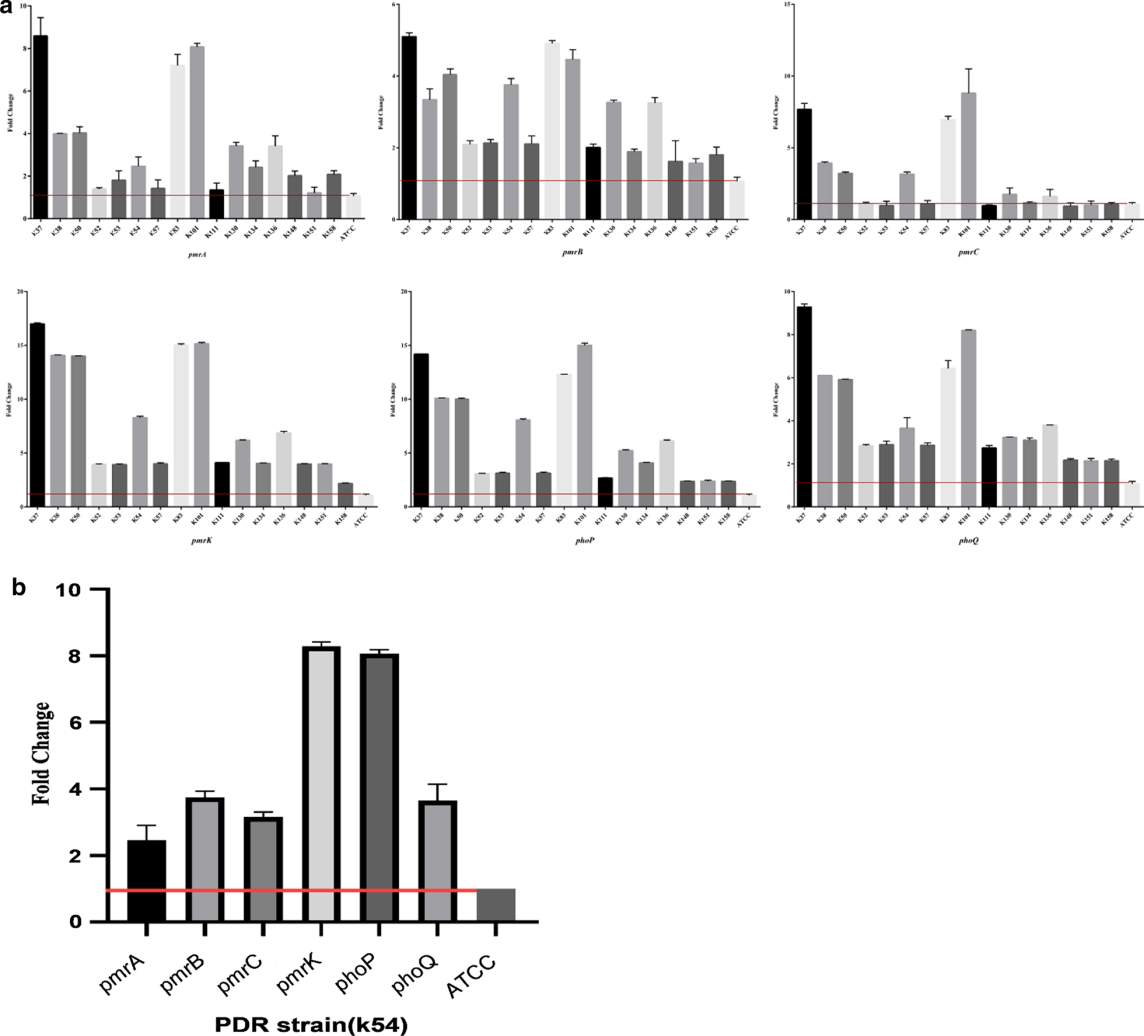
Relative expression levels of the *pmrA*, *pmrB*, *pmrC*, *pmrE*, *pmrD*, *pmrK*, *phoP* and *phoQ* genes in colistin-resistant *K. pneumoniae* isolates. No differences in expression levels were observed for *pmrD* and *pmrE* genes. ATCC: *K. pneumoniae* ATCC 700603. **b** Relative expression levels of the *pmrA*, *pmrB*, *pmrC*, *pmrK*, *phoP* and *phoQ* genes in PDR strain (K54). ATCC: *K. pneumoniae* ATCC 700603

Expression level of *pmr* and *pho* genes was measured to evaluate the effect of mutations on colistin-resistant isolates. Results revealed increased expression level of 1.2–8.6 fold for *pmrA*, 1.57–5.09 fold for *pmrB*, 0.93–8.8 fold for *pmrC*, 2.17–17 fold for *pmrK*, 2.35–15.02 fold for *phoP*, and 2.13–9.28 fold for *phoQ* genes; whereas no differences in expression levels were observed for *pmrD* and *pmrE* genes (Fig. 2a). Analysis of mRNA transcript in K37 isolate with an inactivated *mgrB* gene revealed a significant increase in expression level of genes *pmrA* (8.6-fold), *pmrB* (5.2-fold), *pmrC* (7.3-fold), *pmrK* (17.1-fold), *phoP* (14.5-fold), and *phoQ* (9.3-fold). No insertional inactivation of *mgrB* gene was found in K83 and K101 isolates. Also, features of the colistin-resistant isolates has been showed in Table 5. Relative expression levels of genes in PDR strain shown in Fig. 2b.

**Table 5:** Features of the colistin-resistant isolates

Strain	mRNA relative fold change (mean_SD)											MIC Colistin
	*pmrA*	*pmrB*	*pmrC*	*pmrK*	*phoP*	*phoQ*	*mgrB*	*pmrA*	*pmrB*	*phoP*	*phoQ*	
K37	8/586 ± 0/8623	5/098 ± 0/1077	7/672 ± 0/4285	17/00 ± 0/1000	14/20 ± 1/100	9/283 ± 0/1431	Insertional inactivation, IS5-like element at nt 75	WT	WT	E57 D	WT	128
K38	3/991 ± 1/01,882	3/338 ± 0/3023	3/921 ± 0/1005	14/07 ± 0/04,619	10/07 ± 0/05,196	6/100 ± 0/110	WT	WT	WT	WT	WT	64
K50	4/022 ± 1/2985	4/040 ± 0/1600	3/215 ± 0/09,500	14/00 ± 0/02,887	10/01 ± 0/09,000	5/913 ± 0/02,309	c88t (non-sense, premature termination)	WT	WT	WT	WT	64
K52	1/390 ± 0/06,399	2/090 ± 0/1100	1/100 ± 0/1000	3/933 ± 0/05,774	3/077 ± 0/13,959	2/833 ± 0/05,774	WT	WT	WT	WT	WT	4
K53	1/803 ± 0/4468	2/130 ± 0/1000	0/9667 ± 0/3055	3/927 ± 0/06,429	3/117 ± 0/08,432	2/890 ± 0/1645	WT	WT	WT	WT	WT	4
K54	2/459 ± 0/4448	3/755 ± 0/1750	3/161 ± 0/1489	8/288 ± 0/1324	8/070 ± 0/1127	3/657 ± 0/4841	WT	WT	WT	WT	WT	16
K57	1/423 ± 0/3998	2/105 ± 0/2250	1/100 ± 0/2200	3/993 ± 0/1007	3/114 ± 0/1114	2/863 ± 0/1095	WT	WT	WT	WT	WT	4
K83	7/200 ± 0/5196	4/907 ± 0/08,388	6/950 ± 0/2500	15/03 ± 0/1155	12/31 ± 0/01,732	6/427 ± 0/3719	c88t (non-sense, premature termination)	WT	WT	WT	D150 G	128
K101	8/068 ± 0/1746	4/463 ± 0/2728	8/800 ± 1/700	15/17 ± 0/1155	15/02 ± 0/1921	8/202 ± 0/02,250	c88t (non-sense, premature termination)	WT	T157 P	WT	WT	128
K111	1/341 ± 0/3346	2/010 ± 0/09,000	0/9650 ± 1/0650	4/100 ± 0/010	2/657 ± 0/16,773	2/737 ± 0/1097	WT	WT	WT	WT	WT	4
K130	3/419 ± 0/1695	3/265 ± 0/06,500	1/750 ± 0/4500	6/173 ± 0/04,619	5/212 ± 0/09,789	3/227 ± 0/32,011	c88t (non-sense, premature termination)	WT	WT	WT	WT	8
K134	2/410 ± 0/3100	1/891 ± 0/07,106	1/165 ± 1/06,500	4/033 ± 0/15,774	4/083 ± 0/44,849	3/097 ± 0/1052	WT	WT	WT	WT	WT	4
K136	3/403 ± 0/4935	3/251 ± 0/1506	1/610 ± 0/4900	6/867 ± 0/1528	6/119 ± 0/09,812	3/795 ± 0/009,104	C117a (non-sense, premature termination)	WT	WT	WT	WT	8
K148	2/017 ± 0/2170	1/618 ± 0/5824	0/9343 ± 0/2152	3/980 ± 0/13,464	2/354 ± 0/25,048	2/173 ± 1/06,429	WT	WT	WT	WT	WT	4
K151	1/210 ± 0/2707	1/570 ± 0/1300	1/010 ± 0/2722	3/977 ± 0/14,041	2/387 ± 0/1024	2/137 ± 1/06,429	WT	WT	WT	WT	WT	4
K158	2/082 ± 0/1729	1/801 ± 0/2206	1/043 ± 0/1429	2/173 ± 0/14,619	2/357 ± 1/04,518	2/143 ± 0/1097	WT	WT	WT	WT	WT	4

*K. pneumoniae* ATCC 700603 served as a quality control

*WT* wild type, *nt* nucleotide

### Transformation and conjugation assays

Plasmids carrying *bla*_NDM-1_ and *bla*_NDM-6_ genes in all six strains were successfully transferred to *E. coli* TOPO10 and *E. coli* J53 recipient strains. The antimicrobial resistance profile of the transformants and transconjugants are shown in Table 6. PCR confirmed the presence of the *bla*_NDM-1_ and *bla*_NDM-6_ genes in the transformants and transconjugants; all these isolates harbored also *bla*_CTX-M_*, bla*_TEM_ and *bla*_SHV_ genes (Table 6).

**Table 6:** The features related to NDM-producing *K. pneumoniae* isolates in Iran

Number of isolate	Specimen	Ward	MIC (µg/mL)							ST	Other genes	Plasmid type
			CIP	CTX	CEP	CAZ	IMI	MEM	CO			
K161	Urine	Dialysis	128	32	32	32	128	32	1	147	CTX-M, TEM, SHV	IncL/M
K162	Urine	Dialysis	64	64	64	64	64	64	1	147	CTX-M, TEM, SHV	IncL/M
K165	Urine	Dialysis	128	64	64	64	64	64	1	147	CTX-M, TEM, SHV	IncL/M
K36	Sputum	ICU	128	32	32	32	128	128	1	147	CTX-M, TEM, SHV	IncF
K72	Throat secretions	ICU	128	32	32	32	32	64	1	15	CTX-M, TEM, SHV	IncF
K120	Tracheal tube	ICU	128	512	512	512	16	16	1	3299	CTX-M, TEM, SHV	IncF

### Plasmid replicon typing

Plasmid replicon typing revealed that 3 *bla*_NDM-1_-carrying- and 3 *bla*_NDM-6_-carrying *K. pneumoniae* isolates contained plasmid types belonging to IncF and IncL/M, respectively (Table 6).

### MLST analysis results

STs were identified among the 6 *bla*_NDM_-carrying *K. pneumoniae* isolates, including ST147 (n = 4), ST15 (n = 1), and ST3299 (n = 1). Among the isolates that belonged to ST147, 3 isolates were originated from urine specimens (Table 6).

### Rep-PCR analysis

To evaluate the genetic diversity, 6 *bla*_NDM_-positive and 16 colistin-resistant isolates were subjected to rep-PCR fingerprinting. Isolates were divided into 3 common types (CT) containing 2–4 isolates and 12 single types (ST). Among these, a dominant clone was from Tehran and originated from urine samples. The genotypic pattern of the dominant clone revealed that all isolates harbored ESBL genes.

## Discussion

The excessive and inappropriate use of antibiotics against microbial infections in Iran has led to increased rate of drug resistance in recent decades [36]. Today, clinicians rely increasingly on carbapenems (i.e., imipenem, meropenem, doripenem, etc.) to treat infections due to multidrug-resistant bacteria. CRE strains have been reported in several hospital outbreaks and have the propensity to spread rapidly at local, regional and international levels. The continual emergence of CREs is a major threat to public health worldwide [1]. The worsening condition is that CRE strains show resistance progressively toward a wide range of antimicrobial classes [36, 37] [38]. In this study, about 73.1% of *K. pneumoniae* and 28.3% of *E. coli* isolates were resistant to at least one of the carbapenems tested. Among the included isolates, the highest rates of resistance belonged to piperacillin (n = 161, 97.6%), nalidixic acid (n = 154, 93.3%), and cefotaxime (n = 153, 92.7%). On the other hand, the lowest resistance rate was observed for tigecycline (n = 9, 5.5%) followed by colistin (n = 16, 9.7%), and fosfomycin (n = 26, 15.8%), indicating that these antibiotics have increasingly become primary options for treatment of multi-resistant strains of *K. pneumoniae* and *E. coli*. Our results indicated that the resistance rate of *K. pneumoniae* isolates against colistin was 30.77% with the range MIC 4–128 μg/mL. Colistin remains the last line of defense against many Gram-negative bacilli. However, colistin-resistant and even pan-drug-resistant Gram-negative bacilli have already been reported [39]. According to reports from other studies around the world, the rate of colistin resistance among carbapenem-resistant *K. pneumoniae* has progressively increased from < 2% to 9%. In the last decade in Europe, resistance to colistin has increased to one third of carbapenem-resistant isolates. In addition, multiple outbreaks of colistin-resistant *K. pneumoniae* have been reported in different regions of the world [40, 41].

In this study, the prevalence of ESBL-producing *E. coli* and *K. pneumoniae* were 49.6% and 26.6%, respectively. To date, the ESBL and MBL enzymes has been identified in almost all of the world, including many countries in Asia, Africa, Americas, the Europe, and Australia [42, 43]. The high rate of ESBL and MBL prevalence in the world and its widespread dissemination is a cause of concern. The *bla*_NDM_ are plasmid-mediated genes responsible for resistance to carbapenems and are often co-harbored with different resistance determinants, such as those encoding ESBL. In this study, 98 (59.4%), 54 (32.7%), 77 (46.7%), 3(1.8%) and 3(1.8%) isolates harbored *bla*_TEM_, *bla*_SHV_, *bla*_CTX-M_, *bla*_NDM-1_ and *bla*_NDM-6_ β-lactamase genes, respectively. All three *K. pneumoniae* isolates carrying *bla*_NDM-6_ and one isolate harboring *bla*_NDM-1_ belonged to the ST147 clone. While each of the two remaining isolates that were positive for *bla*_NDM-1_ belonged separately to the ST15 and ST3299 clone. The *bla*_NDM-6_-producing *E. coli* and *K. pneumoniae* have been reported in New Zealand (ST101) [26] and India [44]. Distribution of *bla*_NDM-1_ is greater than that of *bla*_NDM-6_ and was reported from most regions of the world [45, 46].

Plasmids are elements that spread easily. This is one of the most difficult challenges to counteract the dissemination of antibiotic resistance genes and nosocomial infections. Analysis of transformants and transconjugants in the current study revealed that the *bla*_NDM-6_ gene along with *bla*_CTX-M-15_, *bla*_SHV_, and *bla*_TEM_ were carried on transferable plasmids belonging to the IncL/M, while *bla*_NDM-1_ gene was carried on transferable plasmids belonging to the IncF along with *bla*_CTX-M-15_, *bla*_SHV_, and *bla*_TEM_. Previous studies have reported that the spread of *bla*_NDM-1_ is linked to different types of IncA/C, IncF, IncN, and untypeable plasmids [47]. Transferable IncL/M and IncF plasmids have greatly contributed to the dissemination of antibiotic resistance genes, such as *bla*_NDM-6_, *bla*_NDM-1_, *bla*_TEM_, *bla*_SHV_ as well as *bla*_CTX-M-15_ among enterobacterial species [20, 48]. Other study reported that IncL/M and IncF plasmids have the ability to transfer to the susceptible strain, contributing to dissemination of antibiotic resistance genes, such as *bla*_NDM-1_ and *bla*_CTX-M-15_ among *K. pneumoniae* [48, 49]. The three *K. pneumoniae* isolates carrying *bla*_NDM-6_ belonged to ST147, suggesting the possibility of nosocomial infection. ST147 is among the major successful *K. pneumoniae* clone and, usually, is linked to IncF plasmids with *bla*_KPC_ [50].

Colistin is a last-resort antibiotic that has been reintroduced today in clinical practices to treat infections caused by MDR CREs [13]. Acquired resistance to colistin is mostly caused by chromosomal mutations. However, a new plasmid-mediated colistin resistance gene, *mcr*-1, encoding a phosphoethanolamine transferase, has recently been described in China [51]. In our study, plasmid encoded *mcr-1*, *mcr-2*, *mcr-3*, and *mcr-4* genes were not detected in any of the isolates. This results are in line with observations from other studies [29, 52]. Despite low prevalence, various variants of this gene have been reported from different regions of the world, including Iran [53–57]. In addition, many studies have shown the role of chromosomally-mediated mechanisms in colistin resistance [58]. MgrB, a small transmembrane protein with 47 amino acids that regulates the *pmrHFIJKLM* operon through a signaling cascade of PhoPQ, PmrD, and PmrAB and mediates potent negative feedback on the PhoQ/PhoP regulatory system [59]. The insertional inactivation of *mgrB* has been shown to be associated with overexpression of the *phoPQ* and *pmrHFIJKLM* operons, leading to modification of the LPS target, and eventually occurrence of colistin resistance [60]. The insertional inactivation of *mgrB* gene due to IS5-like mobile element was observed in one isolate. In particular, the insertion of IS5-like mobile element at nucleotide 75 of *mgrB* gene was in the same position to that found in other study [30, 52]. Similarly, a truncated MgrB protein by non-sense mutations C88T and C117A was identified in five isolates of the current study, causing premature termination [29, 52]. Remarkably, nine isolates had a wild type *mgrB* gene and also showed no mutations in the other genes associated with resistance to colistin, suggesting the presence of unknown mechanism(s) for colistin resistance. In addition, the mutated PmrB protein, encoded by the *pmrB* gene, is a part of the *pmrCAB* operon, leading to lipopolysaccharide modification and resistance to colistin [31]. In the present study, the A469C mutation in *pmrB* gene led to amino acid substitution Thr157Pro. Jayol et al., identified a Thr residue at position 157, therefore reinforcing the hypothesis that Thr157Pro might play a key role in acquired resistance to colistin [31].

In this study, single–base pair substitutions, including A449G leading to substitution Asp150Gly and A171C leading to substitution Glu57Asp were identified within the *phoQ* and *phoP* sequences, respectively. In other studies, amino acid substitutions in the *PhoQ* gene have been associated with the colistin resistance phenotype Leu26Pro [61], Leu384Gln [62], Asp150Gly [63], Leu96Pro, and Leu348Gln [60]. In *K. pneumoniae*, amino acid substitutions, including Ser85Arg, Thr140Pro, Thr157Pro, Ser205Pro [60] and Thr 157Pro [31] in *pmrB* [62], Leu26Gln and Arg114Ala in *phoP* [60, 63] have been previously reported. In our study, as in Mateur et al., no mutation in the *pmrA* gene was observed [63].

Colistin resistance has been found to be associated with upregulation of *pmrCAB* and *pmrHFIJKLM* operons and *pmrE* gene, resulting in lipidA modification in LPS structure. In this study, the relative expression of *pmrA*, *pmrB*, *pmrC*, *pmrK*, *phoP*, and *phoQ* genes in isolates with *mgrB* mutation (caused by IS element or nonsense mutation) was significantly higher than that of the *mgrB* in wild type isolate and non-mutant colR isolates. In particular, overexpression of studied genes was observed in the *mgrB*-inactivated isolate compared to other isolates. Based on the results of this study and others, increased expression of the genes in *mgrB*-degraded isolates was more noticeable [29–31, 64]. Mutations in *pmrA*/*pmrB* genes resulted in upregulation of the *pmrABC* and *pmrFHIJKLM* operons and *pmrE* gene [31]. The current study revealed an overexpression of the *pmrA*, *pmrB*, *pmrC*, *pmrK, phoP*, and *phoQ* genes in the *pmrB*-mutated isolate compared to that of the *pmrB* gene in wild-type colR *K. pneumoniae*, confirming that the *pmrB* substitution could be responsible for increased expression levels of relevant genes. In the study of Jayol et al., the expression of *pmrA*, *pmrB*, *pmrC*, and *pmrK* genes in isolates with *pmrB*-mutation were significantly increased in comparison with the that of *pmrB* in wild type isolate [31]. Cheng et al., also found Arg256Gly replacement in the *pmrB* in 8 of 26 col-R isolates. All of these eight isolates had overexpressed *pmrHFIJKLM* operon [61].

## Conclusion

The prevalence of carbapenem and colistin resistance isolates among the patients with life-threatening infections hospitalized in critical wards is alarming. Unnecessary prescribing of antimicrobial drugs in patients is associated with the eradication of normal flora, leading to spread of MDR and XDR isolates. The emergence and spread of *bla*_NDM_ and other antibiotic resistance genes in *K. pneumoniae* and *E. coli* will further limit the treatment options and threaten the public health of world.

This study demonstrated that carbapenem and colistin resistance *K. pneumoniae* strains are an emerging threat in different units and should be managed by implementation of timely identification and strict isolation methods that will help to reduce their severe outcomes and mortality rate in critically-ill patients. This study revealed the rapid emergence of extensively-drug resistant *K. pneumoniae* and *E. coli* isolates in patients. In addition, we report for the first time a pan-drug resistant strain from Iran that could be a serious warning for the emergence of highly dangerous strains of nosocomial infections in the future.

The molecular mechanisms investigated in this study found to play a major role in development of resistance to antimicrobials, including carbapenem and colistin. Additional factors, such as increased amount of capsular polysaccharide, efflux pumps, and porins are mechanisms that still needs to be investigated.

## Data Availability

The datasets generated and analyzed during this reasearch were included in the main document of this manuscript.

## Abbreviations

CLSI: Clinical and Laboratory Standards Institute
TSB: Tryptic soy broth
OD: Optical density
PCR: Polymerase chain reaction
RT-qPCR: Quantitative reverse transcription-PCR
MLST: Multi-locus sequencing typing
UTIs: Urinary tract infections
MDR: Multidrug-resistant
CRE: Carbapenem-resistant Enterobacteriaceae
MBL: Metallo-β-Lactamase
rep PCR: Repetitive extragenic palindromic
